# Quantifying variation in forest disturbance, and its effects on aboveground biomass dynamics, across the eastern United States

**DOI:** 10.1111/gcb.12152

**Published:** 2013-02-26

**Authors:** Mark C Vanderwel, David A Coomes, Drew W Purves

**Affiliations:** * Computational Ecology and Environmental Science Group, Microsoft Research21 Station Road, Cambridge, CB1 2FB, UK; 2 Department of Biology, University of FloridaGainesville, FL, 32611, USA; 3 Forest Ecology and Conservation Group, Department of Plant Sciences, University of CambridgeDowning Street, Cambridge, CB2 3EA, UK

**Keywords:** CAIN, carbon fluxes, forest inventory and analysis, hierarchical Bayes, mortality, simulation modeling, tree demography

## Abstract

The role of tree mortality in the global carbon balance is complicated by strong spatial and temporal heterogeneity that arises from the stochastic nature of carbon loss through disturbance. Characterizing spatio-temporal variation in mortality (including disturbance) and its effects on forest and carbon dynamics is thus essential to understanding the current global forest carbon sink, and to predicting how it will change in future. We analyzed forest inventory data from the eastern United States to estimate plot-level variation in mortality (relative to a long-term background rate for individual trees) for nine distinct forest regions. Disturbances that produced at least a fourfold increase in tree mortality over an approximately 5 year interval were observed in 1–5% of plots in each forest region. The frequency of disturbance was lowest in the northeast, and increased southwards along the Atlantic and Gulf coasts as fire and hurricane disturbances became progressively more common. Across the central and northern parts of the region, natural disturbances appeared to reflect a diffuse combination of wind, insects, disease, and ice storms. By linking estimated covariation in tree growth and mortality over time with a data-constrained forest dynamics model, we simulated the implications of stochastic variation in mortality for long-term aboveground biomass changes across the eastern United States. A geographic gradient in disturbance frequency induced notable differences in biomass dynamics between the least- and most-disturbed regions, with variation in mortality causing the latter to undergo considerably stronger fluctuations in aboveground stand biomass over time. Moreover, regional simulations showed that a given long-term increase in mean mortality rates would support greater aboveground biomass when expressed through disturbance effects compared with background mortality, particularly for early-successional species. The effects of increased tree mortality on carbon stocks and forest composition may thus depend partly on whether future mortality increases are chronic or episodic in nature.

## Introduction

Tree mortality is a central process governing the dynamics of forest structure and carbon cycling through terrestrial ecosystems. It is the principle means through which carbon is transferred from live biomass to the soil and atmosphere, and thus serves a critical role in determining whether forest ecosystems act as net carbon sources (Lewis *et al*., 2011) or sinks (Luyssaert *et al*., 2008). Yet, the role of tree mortality in the global carbon balance is complicated by strong spatial and temporal heterogeneity that arises from the ‘slow in – rapid out’ nature of carbon loss through disturbance (Körner, 2003; Fisher *et al*., 2008; Gloor *et al*., 2009). Characterizing spatio-temporal variation in mortality (including disturbance) and its effects on forest and carbon dynamics is thus essential both to understanding the current, substantial carbon sink represented by forests globally (Pan *et al*., 2011), and to predicting how it will change in the future.

Ecologists have long recognized disturbance in particular as an important process shaping the structure and function of forest ecosystems. The classical view of disturbances once held that they acted primarily as catastrophic events that reset forest succession (Clements, 1916). In more recent years, ecologists have viewed the effects of disturbance along a broader continuum (Franklin *et al*., 2002; Turner, 2010). Most forms of disturbance kill only a fraction of all trees present, creating canopy gaps of various sizes (if acting upon large trees), or altering patterns of stand regeneration (if acting upon smaller trees). As a result, disturbances create ecosystem complexity, and are important in promoting species and structural diversity within forest stands (Franklin *et al*., 2002). At the scale of regional and global biogeochemical cycles, disturbances act to transfer carbon from living trees to dead wood, soil, and the atmosphere in the short term, but also alter long-term patterns of carbon accumulation as stand structure and species composition evolve during subsequent recovery (Houghton, 2009).

Although ecologists are beginning to make progress in understanding how various processes contribute to tree mortality (McDowell *et al*., 2011), it remains a considerable challenge to properly integrate the effects of different mortality mechanisms, including disturbance, into terrestrial ecosystem models. Stochastic disturbance processes such as fire, windstorms, disease, or insect outbreaks induce strong variability in forest mortality (Dale *et al*., 2001), leading to instability in carbon sinks over large spatial scales (Coomes *et al*., 2012). The transient, yet often widespread, increases in mortality associated with disturbances need to be distinguished from chronic processes (for example, suppression or senescence) that have predictable influences on the long-term, background mortality risk of individual trees over time. Partly because of such complexities, mortality and disturbance are some of the least well-represented components of terrestrial ecosystem models (Medvigy & Moorcroft, 2012; Smith *et al*., 2012).

Characterizing the nature of forest disturbances is particularly important in the context of global change. Widespread increases in tree mortality have been recently reported across multiple forest regions (van Mantgem & Stephenson, 2007; van Mantgem *et al*., 2009; Peng *et al*., 2011), suggesting that continued warming of the Earth's climate may lead to further increases in mortality rates. A number of potential mechanisms have been identified (Allen *et al*., 2010; McDowell *et al*., 2011), but the available data do not indicate conclusively whether climate-related mortality increases are expected to act through increased background mortality rates (e.g., carbon starvation), through more frequent and intense disturbances (e.g., fire, storms), or through a combination of both. These separate modes of mortality are likely to have differing effects on forest community dynamics and carbon sequestration. For example, an increase in disturbance frequency could stimulate ecosystem productivity by periodically freeing up light and nutrient resources, and by permitting the establishment of faster-growing early-successional species. On the other hand, increased background mortality might act disproportionately on trees that are already weakened or suppressed, with milder overall effects on ecosystem function. Understanding the consequences of these alternative types of mortality increases could help refine predictions of carbon loss and forest compositional changes arising from elevated tree mortality.

With the increasing availability of forest inventory data, researchers have started to investigate the factors that explain broad-scale variation in tree mortality rates for the eastern United States (Lines *et al*., 2010; Dietze & Moorcroft, 2011; Vanderwel *et al*., 2013) as well as other regions (Carnicer *et al*., 2011; Hurst *et al*., 2011). However, to date most of these regional analyses have focused on individual-tree mortality and have not characterized the stand-level variation in tree mortality that results from patchy, stochastic disturbance events (Hurst *et al*., 2011). Past studies of forest disturbance have described or quantified the effects of particular disturbance agents, including fire (Flannigan *et al*., 1998), disease (Voelker *et al*., 2008), insect outbreaks (Hicke *et al*., 2012), or windstorms (Canham *et al*., 2001). Forest inventory data have not yet been used to provide a general quantification of disturbance impacts at a broad scale. By integrating such information within simulation models, we may assess the broader implications of disturbance for regional forest structure and carbon dynamics, and for how they may respond to potential changes in mortality regimes.

Using forest inventory data from the eastern United States, we aimed both to quantify spatio-temporal variation in tree mortality (with a focus on disturbance frequency) and to assess its effects on aboveground biomass dynamics through stand- and regional-scale model simulations. Specifically, we sought to address the following questions. (1) What is the frequency and severity of disturbance-induced mortality for small and large trees in different forest regions across the eastern United States? (2) How persistent is plot-level variation in tree mortality over time? (3) What are the implications of disturbance mortality for stand biomass dynamics across the eastern United States? (4) How do the effects of increased disturbance mortality on forest composition and regional biomass compare with the effects of increased background mortality?

## Materials and methods

### Overview of the CAIN forest dynamics simulator

Our analysis builds on empirical-statistical models for individual-tree growth and mortality that form two parts of the CAIN forest dynamics simulator (Vanderwel *et al*., 2013). The demographic models developed for CAIN were designed to capture general properties of tree growth, mortality, and recruitment in a manner that allows all model parameters to be estimated from forest inventory data and allometric relationships (Caspersen *et al*., 2011). In the version used here, expected tree diameter growth (*G*) and background annual mortality (*M*) rates vary nonlinearly with tree size (diameter at breast height, DBH), height-structured competition for light (vertical profile of crown area index, CAI_h_), and climatic variables (mean annual temperature, MAT, and precipitation, MAP):


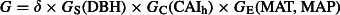
(1)


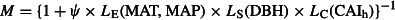
(2)

where *G*_S_, *G*_C_, *G*_E_, *L*_E_, *L*_S_, and *L*_C_ represent non-linear functions, and δ, ψ represent constants (details given in Supporting Information). The functional forms of individual terms were chosen based on successful previous applications in the forest ecology literature (e.g., Coates *et al*., 2009; Lines *et al*., 2010; Caspersen *et al*., 2011; Gómez-Aparicio *et al*., 2011; Hurst *et al*., 2011). Individual terms of the mortality model describe effects on expected longevity (*L*), which we then take the reciprocal of to estimate a tree's annual mortality rate (after first adding one to ensure *M* ≤ 1).

We previously parameterized the models given in Eqn 2 using Forest Inventory and Analysis (FIA) data on tree growth and mortality for seven plant functional types (PFTs) across the eastern United States (Vanderwel *et al*., 2013). More than 200 tree species were classified into six PFTs defined by their latitudinal ranges (boreal, northern temperate, southern temperate) and leaf habit (hardwood, conifer), plus a seventh type (southern temperate hydric) that encompassed species primarily associated with coastal plain swamps. The empirical-statistical models have been proven to accurately capture tree-level variation in growth and mortality.

The growth and mortality models in CAIN form two parts of a cohort-based forest dynamics simulator that can model the structure and PFT-composition of stands across the eastern United States. For any simulated stand, local climate, canopy structure profile, and size determine the background growth and mortality rate of individual tree cohorts of a given PFT. Local climate and canopy structure also determine the number of new individuals of each PFT that recruit into a stand, such that recruitment is higher in more open stand conditions (particularly for shade-intolerant PFTs), as well as in locations where the climate is most suitable for a given PFT. By repeatedly applying these three processes in 5 year timesteps, we can use the demographic models to simulate the long-term (century-to-millenial) dynamics of individual stands and calculate the trajectories of both PFT-specific and total biomass through time. Previous simulations with CAIN have shown that, when run for locations across the eastern United States, the demographic functions produce an emergent geographic distribution for each PFT (i.e., a subset of the region where a given PFT comprises an appreciable part of the community when competing against the others) that approximates its observed distribution well (Vanderwel *et al*., 2013). Simulations with the model also reproduce several key transitions between early- and late-successional PFTs in different parts of this region.

### Quantifying plot disturbance

**Fig. 1:**
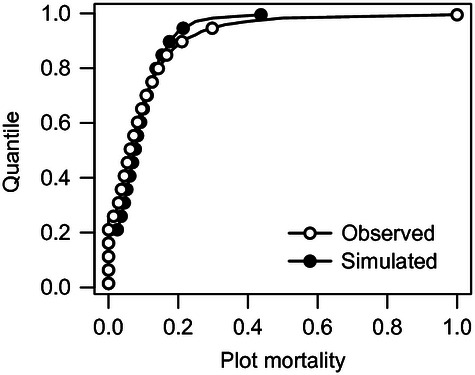
Cumulative frequency distributions for observed plot mortality (fraction of trees that died between plot measurements), and for mortality simulated from individual-level mortality rates predicted by the CAIN model, in 47 723 Forest Inventory and Analysis plots across the eastern United States. Simulated mortality was derived by first drawing a Bernoulli-distributed 1 or 0 value for each tree (given a probability of mortality calculated from its plant functional type, size, crown shading, and climatic environment), then averaging these values at the plot level.

Disturbances are stochastic events that produce large but transient increases in tree mortality within forest stands above the long-term average (‘background’) rate. Although their occurrence cannot be predicted with certainty, it is possible to characterize the frequency and severity of disturbance from tree mortality patterns across a network of re-surveyed forest inventory plots. Within such datasets, disturbances can be recognized within individual plots as conspicuous increases in tree mortality above that predicted from known factors affecting the background rate of mortality, such as tree size, competition, and past environmental conditions. As a preliminary assessment of plot-level disturbance effects, we performed a Monte Carlo simulation of individual tree mortality from the CAIN model (mean mortality rate = 0.0876 for both model and observations), then aggregated the results into a plot-level frequency distribution (Fig. 1). The resulting distribution of simulated plot mortality fails to appropriately capture the upper tail of observed plot mortality. We infer that this upper tail represents the effects of disturbances that kill more than about 25% of trees in a small fraction of plots, but that cannot be predicted based on the tree-level factors accounted for by the model.

We sought to formally quantify the frequency of such mortality events at broad scales. To do so, we extended the CAIN mortality model to estimate the distribution of random plot-level mortality effects, which we define as plot-level increases or decreases in mortality rate compared with the expectations from the original model. To facilitate interpretation, we estimated separate plot effects for small and large trees in two time periods, as well as corresponding effects for tree growth rates (i.e., plot-level increases or decreases in diameter growth rate compared with predictions from the model described above). By examining the joint distributions of these various plot effects for nine distinct forest regions in the eastern United States, we inferred the frequency with which disturbances affect these forests, and how plot-level mortality processes covary between size classes, over time, and with productivity. We then incorporated the estimated variation in mortality in simulations of long-term biomass dynamics to better understand the implications of disturbances for forest composition and carbon storage across the eastern United States.

### Forest inventory and analysis data

We estimated plot-level mortality and growth effects using tree-level remeasurement data from the FIA network of permanent sample plots. The US FIA data have been collected for several decades, and since 1999 have followed a standardized national design in which fixed-radius inventory plots are re-surveyed at 5 year intervals. We downloaded plot data on 26 June 2011 for the eastern United States, including Minnesota, Iowa, Missouri, Arkansas, Mississippi, and all states to the east of these (approximately bounded by the 95th meridian, with the exception of Louisiana, which did not have plots remeasured under the post-1999 national design). We retained the two or three most recent measurements of individual plots that followed the national design. Measurement years ranged from 1995 to 2011 (median = 2002), with 95% of all plots having an interval of 3–8 years between measurements (median = 5).

Each plot consisted of four 7.3 m radius subplots in which all trees ≥12.7 cm DBH were measured, identified to species, and marked as live or dead. Nested within each subplot was a 2.1 m radius microplot in which these same data were collected for all trees ≥2.54 cm DBH. We discarded all subplots that were not on forestland, that were recorded as having been harvested between measurements, that intersected a forest-type condition boundary, or that had a different forest-type condition from the centre of the plot. These data selection procedures left us with repeat measurements for 1 469 606 individual trees ≥2.54 cm DBH on 47 723 plots spanning an area of 3.1 million km^2^.

In estimating plot effects for mortality, we categorized all trees <12.7 cm DBH (i.e., below the subplot size threshold) as ‘small’, and all other trees as ‘large’. We designated plot census intervals that began prior to 2003 as ‘earlier’ intervals, and those that began in or after 2003 as ‘recent’. The dataset contained 9331 plots that were measured successively in ‘earlier’ and ‘recent’ censuses. We also assigned each plot to one of eight regions based on Dyer's (2006) forest region definitions for the eastern United States, or to a ninth ‘Prairie’ region that incorporated plots on the eastern edge of the Interior Plains. Plots in southern Florida that were not part of Dyer's (2006) forest regions were grouped with the Subtropical Evergreen region, which comprises most of the rest of Florida.

In addition to tree-level data, the FIA database contained records for each plot indicating whether it had been disturbed by particular agents (e.g., damage from insects, disease, fire, etc.) since the previous measurement. To qualify, a given disturbance had to affect an area at least 1 acre (0.405 ha) in size and damage or kill at least 25% of trees since the previous plot measurement. We visualized the frequency of recorded disturbances by creating thin plate spline models (each with 100 df) for the geographic distributions of the eight most common disturbance agents, then mapping predictions from these spatial models across the region.

### Estimating plot effects

Here we describe the method by which plot effects were defined and estimated in our analysis of FIA data. For all small or large trees within a given plot, plot effects for mortality add a constant (*E*_M_) to the logit of CAIN's predicted mortality rate (*M*):


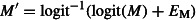
(3)

where the logit function (logit(*x*) = ln(*x*/(1 − *x*))) and its inverse (logit^−1^(*x*) = 1/(1 + exp(−*x*))) translate values on the interval (0,1) to an unbounded real number. As tree mortality rates are typically low (about 1.5% per year) and most mortality effects are near zero, the equation above can be more easily interpreted with the approximation:


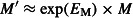
(4)

Plot effects for growth (*E*_G_) are applied as straightforward multiplicative effects on CAIN's predicted growth rate (*G*) for all trees within a given plot:


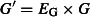
(5)

For each plot in our dataset, we sought to estimate three separate demographic effects for each measurement period: *E*_G_ for tree growth, *E*_M_ for small tree mortality, and *E*_M_ for large tree mortality. Considering both earlier and recent measurement periods, this yielded a total of six effects per plot. We used a multivariate hierarchical Bayesian approach to estimate values for each of these six parameters per plot, and for their joint correlation structure and distribution among plots. In essence, this model-fitting technique determined the values for each mortality and growth plot effect that were most consistent with mortality and growth observations on individual trees within each plot, and also with information that was shared among plots, such as the overall mean and covariance of plot effects within a given forest region.

Formally, the likelihood of each tree's observed mortality (whether it died or not) was calculated from a Bernoulli distribution with a mean of *M*′; the likelihood for observed growth was calculated from a normal distribution with a mean *G*′ and standard deviation σ. The six individual effects for a given plot were assembled into a vector (*v*) that was considered to follow a multivariate normal (MVN) distribution with a mean vector (μ) and a covariance matrix (Σ) that describe the joint distribution of all plot effects within a given forest region. The covariance matrix describes how variable the individual effects are among plots and surveys, as well as the correlations among these effects; e.g., whether elevated mortality in a plot in one survey is associated with elevated (or decreased) mortality in a second survey, or whether elevated mortality is associated with elevated (or decreased) growth. The parameters describing the MVN distribution (μ, Σ) were assigned uninformative priors, and were estimated along with all the plot effects (*v*) and with the parameter for residual error in growth (σ) for each forest region. Parameter inference was performed using the Infer.NET software library (Minka *et al*., 2010) in the C# programming language.

Hierarchical Bayesian analyses are underpinned by a full probability model that can be used to estimate not only the expected mean, but also the posterior probability distribution for each model parameter. By constraining the distribution of plot effects to follow a MVN distribution, we were able to obtain coherent estimates for all plots' mortality and growth effects in both time intervals. Even if little data (e.g., few small or large trees present) or even no data (e.g., plot not measured in the relevant census interval) were available to constrain some effects for a particular plot, the model could still estimate the most likely values for them based on the covariance among the various demographic effects in the other plots.

We note that, like all models, our approach to estimating plot-level demographic variation includes certain assumptions. For example, species-specific disturbances are not likely to be captured particularly well by our plot-centered approach to estimating mortality effects. Although the background model already incorporates differences in expected mortality among PFTs, plot-level deviations from these background rates are applied equally to all individuals and species within a given size category. Also, for simplicity, we have assumed spatial independence among inventory plots. A more complex spatial auto-covariance model might have described whether plots near one another share similar mortality and growth effects, but this was not an important concern at the forest-region scale for which plot-effect distributions were estimated. Thirdly, we have assumed that a joint set of effects for a given plot follows a continuous, well-behaved, and succinct distribution (the multivariate normal). Potential non-linear relationships between the various effects could have been overlooked. No such patterns were apparent in any visual checks of our results, however.

### Simulations

To assess the consequences of variability in mortality effects and disturbance events, we carried out a series of simulations of stand biomass dynamics using the CAIN model. We implemented simulations both with and without dynamic plot mortality effects (hereafter called ‘variable mortality’ and ‘fixed mortality’ scenarios). In simulations with variable mortality, effects for both mortality and growth (*E*_G_, *E*_M_) underwent a correlated random walk within a given stand, varying from one timestep to the next based on the multivariate normal distribution described above (details in Supporting Information). The random walk was correlated because effects for growth or mortality at one time are not independent from those in the recent past. The most important aspect of these simulations was that tree vital rates were subject to the same regime of variation in growth and mortality that we estimated from the inventory data. In simulations with fixed mortality, we held plot mortality effects constant at their expected mean. Growth still varied from one timestep to another, but there was no variation in mortality beyond that implied by the original background mortality model (i.e., mortality rates could still vary because of changes in PFT composition or stand structure, but not because of disturbances).

We used these simulations to understand how variation in mortality and disturbances affect long-term aboveground woody biomass accumulation both at the stand level, and for the eastern United States as a whole. In a first modeling experiment, we simulated one stand per forest region under both the fixed-mortality and variable-mortality scenarios. The simulated stands were initialized with bare ground conditions, and were situated near the centre of their respective forest regions. This experiment enabled us to assess and compare the degree to which disturbances induce variability in stand-level biomass across the eastern United States, relative to stand-level variation in productivity alone.

In a second modeling experiment, we sought to test whether increases in mean tree mortality have different effects depending on whether they act upon background mortality rates, or whether they act via increasing frequency and intensity of disturbance. We carried out simulations in which we artificially modified the distributions of mortality effects to represent different increases in mean mortality rates, from current mortality rates up to a doubling of current rates in 10% intervals. In one set of simulations, we achieved this increase in mean mortality rates by increasing the mode of the mortality-effect distributions (with the variance held constant) to represent an increase in background mortality. In a second set of simulations, we achieved the same mean increase by holding the mode constant and increasing the variance in the mortality-effect distributions to represent an increase in disturbance. We implemented the modeling experiment by taking a random 5% sample of FIA plot locations, running simulations for each of these stands with variability in both growth and mortality effects, and then calculating mean PFT-specific and total biomass across all stands after 500 years. We then compared changes in quasi equilibrium biomass across the gradient of mortality increases, and between the higher-background and higher-disturbance scenarios.

## Results

### Geographic variation in disturbance frequency

**Fig. 2:**
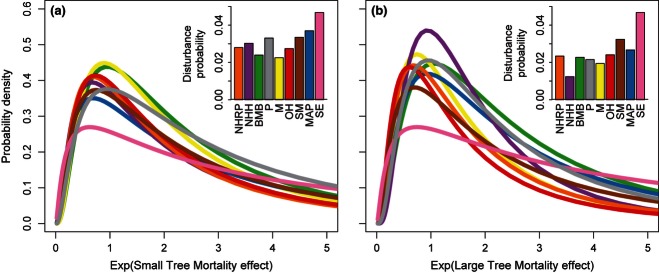
Estimated log-normal distributions of plot mortality effects for (a) small (<12.7 cm DBH) and (b) large trees in nine forest regions within the eastern United States. The horizontal axis corresponds approximately to the expected proportion of the long-term background mortality rate experienced by a given plot in a given census interval. The barplot insets show the probability of experiencing a disturbance event that corresponds to at least a fourfold increase in expected mortality, compared with the long-term plot mean, for each forest region. The full names (acronyms of which are used as labels here) and geographic extents of these forest regions can be found in Fig. 6.

The estimated distributions for plot mortality effects (Fig. 2) revealed that overall rates of disturbance, which we defined as a fourfold increase in plot-level mortality above the expected background rate, were fairly low across the eastern United States (about 3% of all plots affected over approximately 5 years). Large-tree disturbances were least frequent in the northeast (the Northern Hardwoods-Hemlock region), and became increasingly common southwards along the Atlantic coast to the Subtropical Evergreen region. The frequency of disturbances affecting small trees was likewise particularly high in the Subtropical Evergreen region, as well as in the nearby Southern Mixed and Mississippi Alluvial Plain regions. There was a moderate correlation between large and small tree disturbance rates among the nine forest regions (*r* = 0.62 and *r* = 0.78 in the earlier and recent periods, respectively).

**Fig. 3:**
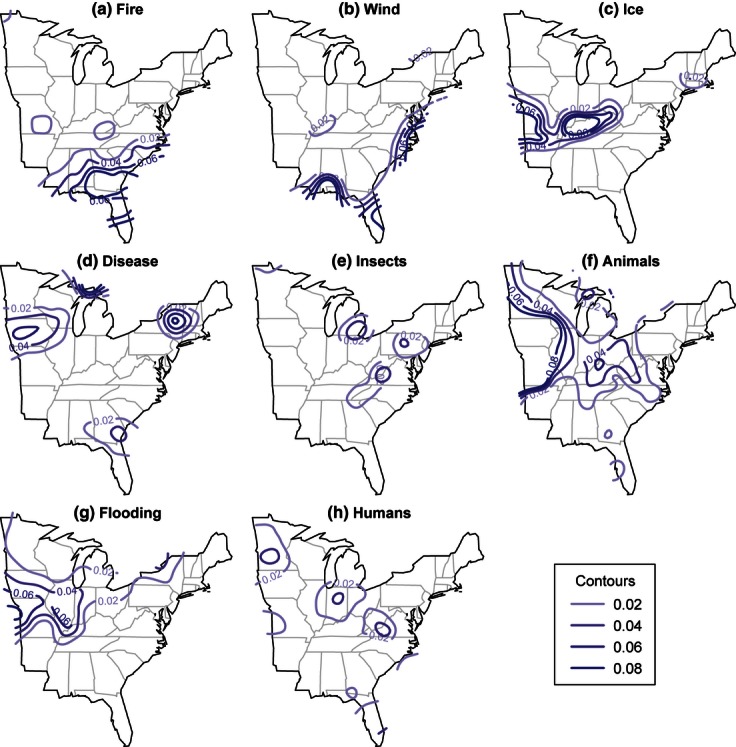
Predicted probability of occurrence for the eight most common forest disturbance agents, as reported during the most recent census of 47 723 Forest Inventory and Analysis plots across the eastern United States (1995–2011). Disturbances are defined here as events that damage or kill at least 25% of trees across an area at least one acre (0.405 ha) in size over since the last plot measurement. Contour lines were generated from the output of thin plate spline models with 100 df (‘Human’ disturbances exclude silvicultural activities).

**Fig. 4:**
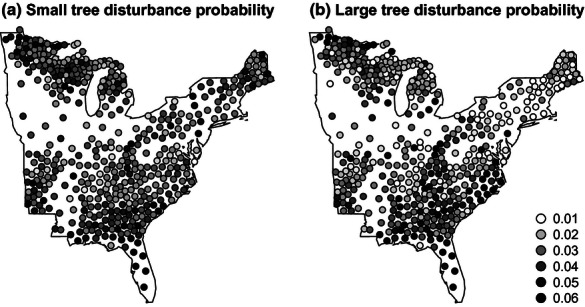
Spatial variation in the estimated frequency of disturbance for small (<12.7 cm DBH) and large trees across the eastern United States. Each circle represents a group of, on average, 100 Forest Inventory and Analysis plots created based on spatial proximity using a *k*-means clustering algorithm. Shading intensity for each circle indicates the mean probability that plots within the cluster had an estimated mortality effect that corresponded to at least a fourfold higher mortality rate than their forest region's mean (the geographic extent of each forest region is shown in Fig. 6).

Records of specific disturbance events in the FIA database provided a clearer picture of how variation in plot mortality effects related to the prevalence of particular types of disturbance (Fig. 3). According to the disturbance records, fire was a major source of disturbance in Southern Mixed and Subtropical Evergreen forests of the Coastal Plain, and mapped closely to the relatively high probability of disturbance within this area (Fig. 4). Wind was a major disturbance along the mid-Atlantic coast, the Gulf coast, and the Florida peninsula; it appeared to explain a large share of disturbance mortality for plots near these coastal areas. Other disturbance agents, including ice storms and insects, did not have any obvious broad-scale spatial relationship with the probability of plot disturbance. Higher incidences of disturbance for both small and large trees along Minnesota's northern border could not be explained by the frequency of records for major plot disturbance events.

**Fig. 5:**
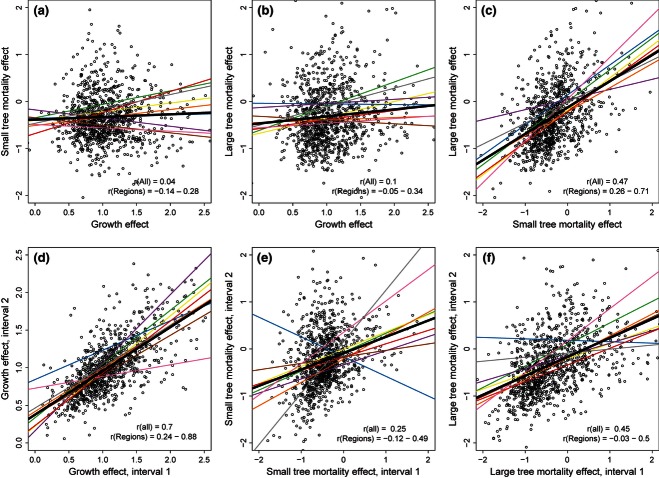
Correlations between plot-level effects for diameter growth, small tree (<12.7 cm DBH) mortality, and large tree mortality (a–c), and correlations in these effects between successive plot measurements (d–f), after accounting for the influence of tree size, competition, and climate in an empirical-statistical model of tree demography. Thick black lines indicate the overall linear relationship between each pair of effects, whereas colored lines indicate relationships within each of nine different forest regions across the eastern United States. The points represent a random sample of 1000 plots (out of up to 47 723 Forest Inventory and Analysis plots) for which these effects were estimated.

The correlation structure for various plot effects showed that plot-level variation in mortality, both for small and large trees, was unrelated to plot-level variation in diameter growth in the same period (*r* = 0.04, 0.10; Fig. 5). Growth effects were likewise unrelated to either small or large tree mortality effects in the previous measurement interval (*r* = 0.05, 0.13). Hence, there was little tendency for elevated mortality to be associated with either increased or decreased tree growth, beyond that accounted for in the background rates. There was a moderate positive correlation between plot mortality effects for small vs. large trees (*r* = 0.47), which was fairly consistent among the different forest regions. Plots that had been remeasured twice over time showed strong correlations in growth effects between two time periods (*r* = 0.70). The persistence of plot mortality effects between measurement intervals was weaker than that of growth, but was still positive for the region as a whole (*r* = 0.25 for small trees; *r* = 0.45 for large trees). Comparing the different forest regions, plot mortality effects in the Mississippi Alluvial Plain region had weak negative correlations between measurement intervals, whereas those in the Subtropical Evergreen region had the strongest positive correlations between intervals.

### Simulations

**Fig. 6:**
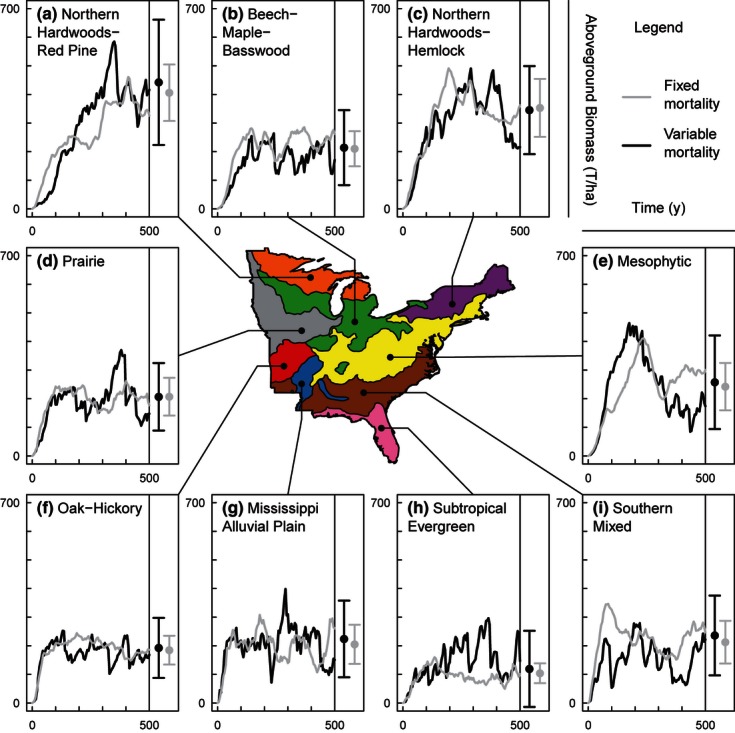
Simulations of aboveground biomass dynamics with the CAIN model for a representative stand in each of nine forest regions across the eastern United States. The black and grey lines in each panel show biomass changes over the first 500 years of each simulation. Error bars on the right of each panel indicate the mean ± 2 SD of aboveground stand biomass for years 500–5000. Aboveground stand biomass tends to exhibit greater variability, but a similar long-term mean, when expected mortality rates vary randomly through time compared with when expected mortality does not fluctuate randomly.

Simulations of stand-level biomass dynamics illustrated some potential consequences of differences in forest disturbance regimes across the eastern United States (Fig. 6). Across most forest regions, stand biomass fluctuated twice as strongly through time in the variable-mortality scenario as compared with the scenario where mortality effects were held fixed. The effects of varying mortality were noticeably different in two regions with the lowest and highest frequencies of disturbance, however. In the Northern Hardwoods – Hemlock region (Fig. 6c), which had the lowest disturbance frequency, stand biomass was only about 50% more variable when the estimated variation in mortality effects was accounted for. In the most frequently disturbed region (Subtropical Evergreen, Fig. 6h), stand biomass was about four times more variable when mortality effects changed through time. In each of the nine forest regions, stand biomass appeared to have a similar long-term mean in both scenarios, regardless of whether disturbances were included.

**Fig. 7:**
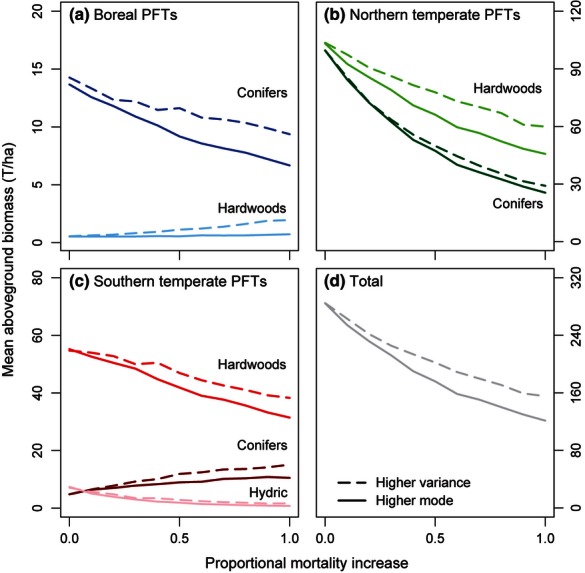
Simulated mean aboveground biomass across eastern United States forests at quasi equilibrium as mortality increases from present levels (proportional increase of 0.0) to double present levels (proportional increase of 1.0). Equivalent increases in mean mortality rates are achieved in one of two ways. Solid lines show the effect of increasing the mode of the estimated log-normal mortality-effect distributions (with constant variance), corresponding to higher rates of chronic background mortality. Dashed lines show the effect of increasing the variance of these log-normal distributions (with constant mode), corresponding to an increase in mean mortality through greater intensities and frequencies of disturbance. All plant functional types represented in the model achieve higher mean aboveground biomass when mortality increases act through intermittent disturbance agents rather than background processes.

Not surprisingly, increases in tree mortality rates were projected to lead to lower biomass for the region as a whole (Fig. 7d). Our simulations further showed that effects would be expressed unequally among different plant functional types (Fig. 7a–c). Late-successional northern temperate hardwoods and conifers showed the largest decreases in biomass in our simulations, but early-successional forest types (boreal hardwoods and southern temperate conifers) actually increased in biomass with elevated mortality rates. Importantly, the process by which mortality increased had a noticeable effect on regional biomass. For a given change in mean mortality, increases in disturbance rates consistently induced smaller decreases (or larger increases) in biomass than increases in background mortality. For example, a doubling of tree mortality through greater disturbance mortality was expected to result in a 45% decrease in total aboveground forest biomass, but a doubling through higher background mortality was expected to decrease total aboveground biomass by 57%. Such differences were particularly pronounced for the early-successional PFTs, which received a disproportionate benefit from increases in the frequency and intensity of disturbances.

## Discussion

Forest disturbances were uncommon across most of the eastern United States. Events that resulted in at least a fourfold increase in tree mortality (over a time interval of about 5 years) were observed in only 1–5% of plots in different forest regions (Fig. 2). Catastrophic mortality events (i.e., death of virtually all trees) were rarer still, occurring in only about 0.5% of all plots (Fig. 1). On the whole then, current forest dynamics in the eastern United States appear not to be driven by natural disturbances to the same extent as some other forest regions (Mouillot & Field, 2005). Nevertheless, our analyzes teased out considerable variability in the causes and effects of disturbance across this area. We discovered that geographic variation in disturbance frequency can induce important qualitative differences in the biomass dynamics of northeastern and southern forests, with variation in mortality causing the latter to undergo four times larger fluctuations in stand biomass than would be the case if only growth varied through time. We have also shown that if mortality rates increase due to environmental change, most forest types across this region could express discernibly different responses depending on whether the increase came from higher background mortality, or from more frequent and intense disturbances.

### Regional variation in disturbance processes

We estimated that disturbance rates were highest in the three southernmost forest regions (Southern Mixed, Mississippi Alluvial Plain, and Subtropical Evergreen). By no coincidence, these regions largely overlapped with the distributions of both fire and windstorms, the two most commonly recorded disturbance agents in the southern United States. Many pine- and oak-dominated stand types in southern forests are prone to regular understory and mixed-severity fire, which have historically occurred with a frequency of <10 years (Wade *et al*., 2000). Coastal areas in these southern regions are also susceptible to strong hurricane damage. Hurricanes were particularly common within these areas in the years 2004–2005 (Changnon, 2009), and were responsible for considerable tree mortality during this time. Hurricane Katrina alone was estimated to kill 320 million trees within Gulf Coast forests (Chambers *et al*., 2007).

Although ice storms occur most frequently in the northeastern United States (Irland, 2000), the plot disturbance records predominantly reflected a 2009 ice storm centered in Kentucky and Arkansas (Haavik *et al*., 2012). This storm had a detectable, though not especially strong, relationship with our estimated mortality effects in the Mesophytic and Oak-Hickory forest regions. Ice storms are known to cause considerable crown damage but, generally speaking, are only believed to cause widespread mortality in cases where heavy glazing results in at least 75% crown loss (Proulx & Greene, 2001).

Mortality effects in northern and central forest regions appeared to be locally related to insects and disease, but otherwise did not show obvious relationships with recorded disturbances. The disjunct distributions of insects and disease corresponded with specific pests and pathogens, including beech bark disease in the northeast, Dutch elm disease in the midwestern prairie, and the Hemlock woolly adelgid along the Appalachians (Lovett *et al*., 2006; Hicke *et al*., 2012). Eastern spruce budworm and forest tent caterpillar may also be responsible for higher disturbance frequencies in northern Minnesota (Robert *et al*., 2012), although damage from these particular insects was not widely noted in disturbance records.

The probability of disturbance across northern and central parts of the eastern United States is much more diffuse than the distributions of these biotic agents, suggesting that other processes account for many disturbances across this area. In particular, windstorms are a dominant agent of disturbance across this broad region (Frelich & Lorimer, 1991; Peterson, 2000; Canham *et al*., 2001; Schulte & Mladenoff, 2005; Hanson & Lorimer, 2007). Thunderstorm downbursts and hurricanes, both of which occur with variable intensity, are the most common sources of wind mortality (Peterson, 2000). While catastrophic blowdowns do occur with very long return intervals, both our analysis and previous studies (Frelich & Lorimer, 1991) indicate that mild to moderate wind mortality across the wider forest landscape is a more important disturbance process to the structure and dynamics of these forests.

### Size, productivity, and temporal relationships of plot mortality effects

Tree size is related to susceptibility to most mortality and disturbance processes. A moderate correlation between mortality effects for small and large trees would seem to reflect that, overall, mortality processes that strongly affected large trees affected small trees somewhat less, and *vice versa*. For example, surface fires predominantly kill smaller trees but can also kill larger ones (Beverly & Martell, 2003), whereas windstorms are more likely to snap or uproot trees in the canopy than the understory (Canham *et al*., 2001). The size correlation was weakest in the Northern Hardwood-Hemlock region, which experienced less large tree disturbance than anywhere else. Disturbance processes that affect distinct life history stages (such as sapling herbivory) would presumably operate more independently across size classes, but our model did not include data for trees <2.5 cm DBH.

Unlike growth, correlation between mortality effects over time was quite modest. The lack of strong temporal persistence indicates that mortality effects are not predominantly driven by differences in edaphic conditions, variation in species composition within each PFT, bias in the underlying CAIN mortality model, or other lasting differences between plots. The estimated temporal correlations do, however, reveal that plot mortality is not completely independent from 1 to 5 year interval to the next. Some mortality processes, such as drought or insect defoliation, can operate over a period of several years. In other cases, tree death can occur as a lagged response to particular stressors (Franklin *et al*., 1987). Hence, although the expected mortality rate for a given plot may vary strongly over time, it is still partly related to the mortality rate in the recent past.

We have invoked a space-for-time substitution (Pickett, 1989) in inferring distributions of disturbance frequency and intensity from plot-to-plot variation in mortality effects. One would like assurances that an indirect method such as this is sound. Fortunately, the estimated covariance structure of plot demographic effects lets us assess whether our approach and results provide a reasonable quantitative description of variation in mortality through time. Firstly, we note that neither small nor large tree mortality effects were related to stand productivity. Several hypotheses can explain relationships between mortality and productivity (Stephenson *et al*., 2011). For example, growth and mortality may covary along edaphic gradients, or there may be inter-specific trade-offs between maximum growth rate and survival. Predicted growth and mortality were negatively correlated in CAIN's empirical-statistical models, because greater crown shading both decreased growth rate and increased the probability of mortality for individual trees. However, the fact that no such relationships were present in our plot-level demographic effects (after controlling for the effects of PFT, size, competition, and climate in CAIN's demographic models) strongly suggests that stochastic processes are affecting mortality independently of growth. Secondly, mortality effects for small and large trees were correlated with one another both across and within forest regions. If mortality effects were the result of demographic stochasticity – that is, if many trees happened to die in a given plot simply by random chance – then consistent, positive correlations between size classes would not be expected. Thirdly, there was substantial variation in mortality effects within a given plot from one time period to the next. Growth effects were largely persistent over time, but mortality effects for both small and large trees varied much more strongly between successive census intervals. This variability indicates that transient or fluctuating processes dominate plot-level mortality dynamics. In the future, additional plot censuses could help to more explicitly resolve spatial and temporal components of variation in plot mortality.

### Implications for stand- and region-level biomass dynamics

Previous stand- and landscape-level modeling studies have shown that particular disturbance processes can have important effects on species composition (Tremblay *et al*., 2005; Papaik & Canham, 2006; Uriarte *et al*., 2009), short-term carbon flux (Masek & Collatz, 2006; Desai *et al*., 2007), and long-term biomass accumulation (Scheller & Mladenoff, 2005) in forests. Still, it has proven to be a considerable challenge to appropriately scale up processes, data, and models for disturbance from local sites up to broader regions (Liu *et al*., 2011; Coomes *et al*., 2012). In this study, we developed an approach that estimated variation in mortality rates from regional inventory data, then incorporated these effects within a stand-level forest dynamics simulator that can be applied anywhere in the region. By implementing correlated random walks for demographic effects within the CAIN simulation model, we were able to assess the overall consequences of temporal variation in mortality for stand biomass dynamics across the eastern United States.

This kind of empirical quantification of mortality complements bottom-up attempts at predicting tree mortality from ecophysiological processes (McDowell, 2011). The ecophysiological approach is potentially valuable because, unlike empirically based studies such as ours, it may be able to address the direct or indirect effects of increasing CO_2_ on mortality. However, the fact that trees can die in many ways that are only weakly related to ecophysiological processes limits the extent to which tree mortality can ever be predicted in this manner alone. A combination of both approaches could provide a much better understanding of the role of tree mortality in determining the response of the world's forests to climate change.

According to our simulation results, fluctuations in aboveground stand biomass are generally about twice as great when variation in mortality effects is considered. The observed geographic gradient in disturbance frequency implies that Subtropical Evergreen forests should exhibit particularly strong biomass variation due to disturbance, whereas in the northeast, most variation in biomass may be attributable to changes in productivity. (Although we did not implement any stochastic variation in recruitment, preliminary investigations indicated that simulation output was relatively insensitive to changes in recruitment.) The qualitative differences in biomass dynamics that we report could have a variety of ecological and evolutionary consequences, including a regional north-south gradient in life-history traits and tree species richness (Loehle, 2000). Disturbance-driven biomass dynamics may also help explain, for example, why southern forests are dominated by early-successional pines and mid-successional oaks, but many northern forests are dominated by late-successional maples or balsam fir (*Abies balsamea*).

Our regional-scale simulations further illustrate how changes in mortality regimes can affect aboveground biomass across thousands of stands sampled from the entire eastern United States. For all seven different plant functional types considered, we found that a given long-term increase in mortality would lead to higher biomass when expressed through disturbance effects compared with chronic background mortality. The relative difference was most pronounced in early-successional PFTs such as boreal hardwoods and southern-temperate conifers. Moderate- and high-intensity disturbances create open stand conditions and large pulses of subsequent recruitment, particularly for early-successional PFTs. Over the following years, stands dominated by smaller, younger, and generally less shade-tolerant trees would have lower hydraulic resistance, greater growth efficiency, and higher overall productivity than undisturbed stands (Caspersen *et al*., 2011). Consequently, future increases in disturbance rates could induce changes in both stand structure and composition that would help mitigate decreases in mean aboveground biomass at a regional scale.

Although, future climate change is expected to lead to increased forest mortality (van Mantgem & Stephenson, 2007; van Mantgem *et al*., 2009; Peng *et al*., 2011), our current understanding of mortality processes is not sufficiently developed to predict the amount or the manner by which mortality rates might increase. For example, drought stress is expected to become more common under projected changes in temperature and precipitation (Allen *et al*., 2010). This might lead to a larger fraction of all trees dying each year as water becomes more limiting overall, or it might lead to more frequent large-scale forest dieback in years of extreme drought. Unfortunately, mechanistic models are not yet adequate for predicting the magnitude of drought-induced mortality, nor how it will vary in space and time. Potential future changes in other mortality agents – windstorms, fire, and insect pests for example – are even more uncertain. Our results show that the implications for terrestrial ecosystems and carbon storage will depend, to a good extent, on how such changes affect both the mean and the spatio-temporal variance in tree mortality.

With increasing availability of inventory data for many of the world's forests, it may be possible to extend the approach presented here to estimate how both background mortality and disturbance vary with climate, plant functional type, individual tree size, and other factors, wherever such data are available. This could lead to an ability to simulate plausible future scenarios for background mortality and disturbance more widely, and to thereby identify regions where forest carbon is most vulnerable to environmental change at a global scale. Assessments of forest mortality risk that are informed by data-driven models of forest dynamics would greatly help in predicting the future of the global carbon sink, and in guiding attempts to protect forest carbon through incentive-based conservation programs (Miles & Kapos, 2008).

## Supporting Information

**Appendix S1.** Details of CAIN forest dynamics model.

**Appendix S2.** Simulating dynamic mortality and growth effects.

**Appendix S3.** Simulating increased background and disturbance mortality.
