# Supplementary material for: Quantifying variation in forest disturbance, and its effects on aboveground biomass dynamics, across the eastern United States
Source: Glob Chang Biol. 2013 Feb 26;19(5):1504–17. doi: 10.1111/gcb.12152 (PMC3657128; doi:10.1111/gcb.12152)
Supplement: Supplementary file 1 [file gcb0019-1504-SD1.docx]

Supporting Information for Vanderwel et al. “Quantifying variation in forest disturbance, and its effects on aboveground biomass dynamics, across the eastern United States”

Appendix A. Details of CAIN Forest Dynamics Model

A.1 Tree Growth and Mortality

For each plant functional type (PFT), annual tree diameter growth (*G*, cm·y^-1^) and mortality rates (*M*, y^-1^; the inverse of longevity, *L*) were modelled as the product of a maximum rate term (*δ*, *ψ*) and three terms that account for the effects of tree size, local competition, and environmental conditions (mean annual temperature, *MAT* (°C), and precipitation, *MAP* (cm·y^‑1^)):

$$G=\delta\times G_{S}(DBH)\times G_{C}({CAI}_{h})\times G_{E}(MAT,MAP)$$

 (A1)

$$M=\left\{ 1+\psi\times L_{E}\left( MAT,MAP \right)\times L_{S}\left( DBH \right)\times L_{C}\left( {CAI}_{h} \right) \right\}^{-1}$$

 (A2)

*Size effects*: To account for the effect of tree size, growth varies as a log-normal function of diameter, whereas longevity both increases as a power function of diameter and decreases as a sigmoidal function of diameter to produce an overall U-shaped mortality function:

$$G_{S}\left( DBH \right)=\exp\left\{ -0.5\times\left[ {ln(DBH/\gamma)}/\nu\right]^{2} \right\}$$

 (A3)

$$L_{S}\left( DBH \right)=\frac{\left( {DBH}/{10} \right)^{\phi}}{1+\text{exp}\left[ \lambda\times\left( DBH-\theta\times{DBH}_{0.01} \right) \right]}$$

 (A4)

where *γ*, *ν*, *ϕ*, *θ*, and *D_0.01_* are PFT-specific parameters and

$$\lambda=\ln\left( 99 \right)/\left[ D_{0.01}\times(1-\theta) \right]$$

 (A5)

*Competition effects*: Height-structured competition is implemented through a state variable for a plot’s crown area index (*CAI_h_*), a unitless measure for the projected area of all tree crowns (*CA_h,i_*, m^2^) at a given height *h* (m), normalized by the area of the plot (*A*, m^2^):

$${CAI}_{h}=\frac{\sum_{i}^{N} {CA}_{h,i}}{A}$$

 (A6)

Projected crown areas were calculated based on the tree height and crown allometry models described in Purves et al. (2007) and in Vanderwel et al. (2012). Projected crown area remains constant from the base of a tree’s crown to the ground, so *CAI_h_* increases montonically from the top of the canopy to the ground.

To account for the effect of competition, both growth and longevity decrease as negative exponential functions of *CAI_h_*:

$$G_{C}\left( {CAI}_{h} \right)=\zeta+\left( 1-\zeta\right)\times\exp\left( -\kappa\times{CAI}_{h} \right)$$

 (A7)

$$L_{C}\left( {CAI}_{h} \right)=\omega+(1-\omega)\times exp(-o\times{CAI}_{h})$$

 (A8)

where *ζ*, *κ*, *ω*, and *ο* are PFT-specific parameters and *CAI_h_* is evaluated at the mid-crown height, *h* (m), of the tree.

*Environmental effects*: To account for the effects of climate, we first defined the major environmental axes of variability in growth and mortality (*E_G_, E_L_*) as linear combinations of mean annual temperature and precipitation. For each vital rate, this climate axis was defined by a single parameter (*g_1_*, *l_1_*) that determined whether performance varied most strongly with a spatial gradient in temperature, with precipitation, or with some combination of temperature and precipitation:

$$E_{G}=\cos\left( g_{1} \right)\times MAT'+sin(g_{1})\times MAP'$$

 (A9)

$$E_{L}=\cos\left( l_{1} \right)\times MAT'+sin(l_{1})\times MAP'$$

 (A10)

where *g_1_* and *l_1_* are PFT-specific parameters, and *MAT′* and *MAP′* are mean annual temperature and precipitation values that have been rescaled to the range [0, 1]. We rescaled *E_G_* and *E_L_* to the range [0,1] for each PFT, and allowed growth and mortality to vary as Gaussian functions of these climate gradients:

$$G_{E}\left( MAT, MAP \right)=exp\left[ {-\left( \frac{{E'}_{G}-g_{2}}{g_{3}} \right)}^{2} \right]$$

 (A11)

$$L_{E}\left( MAT, MAP \right)=exp\left[ {-\left( \frac{{E'}_{L}-l_{2}}{l_{3}} \right)}^{2} \right]$$

 (A12)

where *g_2_*, *g_3_*, *l_2_*, and *l_3_* are PFT-specific parameters, and *E′ _L_* and *E′ _G_* represent positions along the rescaled climate gradient. Ranging for *MAT*, *MAP*, *E_G_*, and *E_L_* introduced additional calculations, but greatly aided parameter fitting by ensuring that the variables were always bounded by 0 and 1.

A.4 Recruitment

Annual recruitment for each PFT (*I*, ha^-1^·y^-1^) was defined as the density of stems, per year, that reach the minimum DBH threshold used in our simulations (3 cm). Recruitment was modelled as the product of a maximum rate term (*τ*) and three terms that account for the effects of local competition, landscape-level propagule sources, and environmental conditions (*MAT*, *MAP*):

$$I=\tau\times I_{C}({CAI}_{0})\times I_{L}(LBA)\times I_{E}(MAT,MAP)$$

 (A13)

where *LBA* (m^2^·ha^-1^) is the basal area of the PFT in the landscape surrounding a given plot.

*Competition effects*: Like growth and mortality, recruitment decreases as a negative exponential function of crown area index. For recruitment, *CAI_h_* is evaluated at the level of the ground (*h*=0). We modified the negative exponential function to have a flat shoulder up to a certain value of *CAI_0_*, above which estimated recruitment begins to decrease:

$$I_{C}\left( {CAI}_{0} \right)=min\left\{ 1,\chi+(1-\chi)\times exp\left[ -\upsilon\times({CAI}_{0}-i_{5}) \right] \right\}$$

 (A14)

where *χ*, *υ*, and *i_5_* are PFT-specific parameters.

*Source effects*: The CAIN model accounts for variation in recruitment with the abundance of the PFT in the surrounding area by allowing recruitment to increase as a power function of *LBA*:

$$I_{L}={(LBA/5)}^{i_{4}}$$

 (A15)

where *i_4_* is a PFT-specific parameter. For the purposes of the simulations presented here, we fixed *LBA* for each PFT to the global constant of 5.66, which was the average basal area of each PFT in counties for which a given PFT had a mean abundance of at least one tree per plot. This had the effect of removing landscape-level source effects on recruitment within our simulations.

*Environmental effects*: The effects of climate on recruitment were modelled in the same manner as they were for growth and mortality.

$$E_{I}=\cos\left( i_{1} \right)\times MAT'+sin(i_{1})\times MAP'$$

 (A16)

$$I_{E}\left( MAT, MAP \right)=exp\left[ {-\left( \frac{{E'}_{I}-i_{2}}{i_{3}} \right)}^{2} \right]$$

 (A17)

where *i_2_*, *i_3_*, and *i_4_* are PFT-specific parameters.

References

Purves DW, Lichstein JW, Pacala SW (2007) Crown plasticity and competition for canopy space: a new spatially implicit model parameterized for 250 North American tree species. *PloS ONE*, **9**, e870.

Vanderwel MC, Lyutsarev V, Purves DW (2012) Climate-related variation in mortality and recruitment determine regional forest-type distributions. *Global Ecology and Biogeography*, in review.

Appendix B. Simulating Dynamic Mortality and Growth Effects

We used our estimated plot-effect distributions for different forest regions to simulate correlated random walks in stand-level mortality and growth effects over time. Here we provide mathematical details on how plot effects were updated for each stand from one timestep to the next.

Firstly, for each forest region we calculated the mean (μ) and standard deviation (σ) for growth effects, small tree mortality effects and large tree mortality effects from the pooled set of estimates across both earlier and recent census intervals in the FIA plots. These values were assembled into a mean vector (μ_0_) and diagonal standard deviation matrix (**S_0_**):

$$\mu_{0}=\left[ \begin{matrix} \mu_{G} \\ \mu_{SM} \\ \mu_{LM} \end{matrix} \right]$$

(B1)

$$\boldsymbol{S}_{\boldsymbol{0}}\boldsymbol{=}\left[ \begin{matrix} \sigma_{G} & 0 & 0 \\ 0 & \sigma_{SM} & 0 \\ 0 & 0 & \sigma_{LM} \end{matrix} \right]$$

(B2)

We also estimated correlations (ρ) between each pair of demographic effects, and assembled these into a correlation matrix (**R_0_**):

$$\boldsymbol{R}_{\boldsymbol{0}}=\left[ \begin{matrix} 1 & \rho_{G\cdot SM} & \rho_{G\cdot LM} \\ \rho_{G\cdot SM} & 1 & \rho_{SM\cdot LM} \\ \rho_{G\cdot LM} & \rho_{SM\cdot LM} & 1 \end{matrix} \right]$$

(B3)

We then created another matrix (**R_1_**) that contained the correlations among demograhic effects between successive census intervals. The elements of this matrix were obtained directly from the forest region-specific covariance matrices that were estimated from the FIA data:

$$\boldsymbol{R}_{\boldsymbol{1}}=\left[ \begin{matrix} \rho_{G1\cdot G2} & \rho_{G1\cdot SM2} & \rho_{G1\cdot LM2} \\ \rho_{SM1\cdot G2} & \rho_{SM1\cdot SM2} & \rho_{SM1\cdot LM2} \\ \rho_{LM1\cdot G2} & \rho_{LM1\cdot SM2} & \rho_{LM1\cdot LM2} \end{matrix} \right]$$

(B4)

Next, we used these components to create a new six-dimensional multivariate normal distribution, *N*(μ, **V**), that described the joint distribution of plot effects within and between timesteps of our simulations. The first three dimensions represented plot-effects for growth, small tree mortality, and large tree mortality in a given five-year time interval; the second three dimensions represented these same effects in the following five-year interval. The mean vector (μ) and covariance matrix (**V**) of this multivariate normal distribution were created from μ_0_, **S_0_**, **R_0_**, and **R_1_** as follows:

$$\mu=\left[ \begin{matrix} \mu_{0} \\ \mu_{0} \end{matrix} \right]$$

(B5)

$$\boldsymbol{S}=\left[ \begin{matrix} \boldsymbol{S}_{\boldsymbol{0}} & 0 \\ 0 & \boldsymbol{S}_{\boldsymbol{0}} \end{matrix} \right]$$

(B6)

$$\boldsymbol{R}=\left[ \begin{matrix} \boldsymbol{R}_{\boldsymbol{0}} & \boldsymbol{R}_{\boldsymbol{1}} \\ {\boldsymbol{R}_{\boldsymbol{1}}}^{'} & \boldsymbol{R}_{\boldsymbol{0}} \end{matrix} \right]$$

(B7)

$$\boldsymbol{V}=\boldsymbol{SRS}$$

(B8)

We carried out model simulations under two scenarios: one in which growth effects changed from one timestep to another but mortality effects were fixed at their overall mean (“fixed mortality”); and a second in which demographic effects for both growth and mortality changed through time (“variable mortality”). Both scenarios were implemented by updating demographic effects on a stand-by-stand basis by drawing conditional samples from the multivariate normal distribution described above.

Conditional samples can be drawn from a multivariate normal distribution by partitioning the mean vector and covariance matrix into those dimensions that are fixed (subscripted with 2 below) and those that are described probabilistically (subscripted by 1):

$$\mu=\left[ \begin{matrix} \mu_{1} \\ \mu_{2} \end{matrix} \right]$$

(B9)

$$\boldsymbol{\Sigma}=\left[ \begin{matrix} \boldsymbol{\Sigma}_{11} & \boldsymbol{\Sigma}_{12} \\ \boldsymbol{\Sigma}_{21} & \boldsymbol{\Sigma}_{22} \end{matrix} \right]$$

(B10)

The distribution of x_1_ conditional on x_2_ = a then follows a multivariate normal distribution:

$$\Pr\left( x_{1} | x_{2}=a \right)\sim N(\bar{\mu},\bar{\boldsymbol{\Sigma}})$$

(B11)

where:

$$\bar{\mu}=\mu_{1}+\boldsymbol{\Sigma}_{12}{\boldsymbol{\Sigma}_{22}}^{-1}(a-\mu_{2})$$

(B12)

$$\bar{\boldsymbol{\Sigma}}\boldsymbol{=}\boldsymbol{\Sigma}_{11}\boldsymbol{-}\boldsymbol{\Sigma}_{12}{\boldsymbol{\Sigma}_{22}}^{-1}\boldsymbol{\Sigma}_{21}$$

(B13)

We used these properties to simulate correlated random walks in growth and mortality effects over time. In the fixed mortality scenario, we initialized the simulation by fixing all mortality effects to values representing mean levels of mortality, then made a conditional random draw for the remaining growth effects. In each subsequent timestep, we drew a new growth effect conditional on the current growth effect and mean mortality effects. We then set the current growth effect to this new value and applied it in the demographic model for diameter growth (G') for all trees in the stand.

In the variable mortality scenario, we followed a similar procedure but did not fix mortality effects to their mean values. Thus, both growth and mortality effects were updated over time by drawing a new set of values each timestep that were conditional upon the current set of values. The new values were then applied to modify both growth and mortality rates (G', M') of all trees present.

Appendix C. Simulating increased background and disturbance mortality

In our second simulation modelling experiment we increased mean mortality rates from current values up to double current values, in increments of 10%. These increases in mean mortality rates were achieved either by increasing the mode of the mortality effect distribution (representing higher background mortality rates) or by increasing the variance of the mortality effect distribution (more frequent and intense disturbances). Here we describe how we modified the estimated mortality distributions to implement these changes.

Plot mortality effects (*E_M_*) alter modelled tree-level probabilities of mortality (*M*) according to eq. 2 of the main text:

$$M^{'}={logit}^{-1}(logit\left( M \right)+E_{M})$$

(C1)

The joint set of plot effects (growth and two mortality size classes, each in two census intervals) follows a multivariate normal distribution. Focussing on just one mortality effect, the marginal distribution of each *E_M_* follows a normal distribution, which is then translated into new tree-level mortality rates (*M'*) via the logit function. For a given value of *M*, the normal (marginal) distribution of *E_M_* thus produces a logit-normal distribution (Frederic and Lad 2008) for *M'*.

For our simulations of increased mortality, we wished to change either the mean (μ) or standard deviation (σ) of the marginal distribution of *E_M_* such that, for a typical value of *M*, *M'* = *b*·*M*, where *b* is one of {1, 1.1, 1.2, …, 2.0}. As there is no analytical solution for the mean of a logit-normal distribution, we used a sampling-based approach to estimate the appropriate change in μ or σ for each forest region and level of mortality increase (*b*). We based our calculations on a value of *M* = 0.0139, which was the average annualized biomass turnover rate across the eastern US when simulations were run to quasi-equilibrium with variable mortality effects. Using a local optimization routine, we searched for a new value of either μ or σ for which:

$$\frac{E(M^{'})}{M}-b=0$$

(C2)

where E(*M'*) is the expected value of *M'* obtained by integrating over the new candidate (marginal) distribution of *E_M_*. In all cases, we retained the original value of the parameter that was not changed (i.e., we increased background mortality by changing μ but not σ, and we changed disturbance rates by changing σ but not μ).

Reference

Frederic P, Lad F (2008) Two moments of the logitnormal distribution. *Communications in Statistics – Simulation and Computation*, **37**, 1263-1269.
